# Depression and thoughts of self‐harm and suicide among people living with dementia: results of a cross‐sectional survey

**DOI:** 10.1111/psyg.12996

**Published:** 2023-06-21

**Authors:** Mariko Carey, Elise Mansfield, Emilie Cameron, Allison Boyes, William Browne, Jason Dizon, Rob Sanson‐Fisher

**Affiliations:** ^1^ School of Medicine and Public Health, College of Health and Wellbeing University of Newcastle Callaghan New South Wales Australia; ^2^ Hunter Medical Research Institute New Lambton Heights New South Wales Australia; ^3^ Eastern Health, Maroondah Hospital Melbourne Victoria Australia

**Keywords:** Alzheimer's disease, cross‐sectional studies, dementia, depression, suicidal ideation, unmet needs

## Abstract

**Background:**

Depression is common among people with dementia. Despite most people with dementia living in the community, there have been few investigations of self‐reported depressive symptoms and suicidal ideation among community‐dwelling people with dementia in Australia. This study aimed to explore the proportion of people with mild, moderate and severe levels of depressive symptoms, and suicidal ideation among a sample of people living with dementia in Australia. Correlates of reporting depressive symptoms were also explored.

**Methods:**

Adults diagnosed with dementia by a medical professional who were English speaking and community‐dwelling were asked to complete a paper and pencil survey. Those who were unable to provide independent consent were excluded. Depression was assessed using the Geriatric Depression Scale −15, and suicidal ideation was assessed using two study‐specific items. Multivariable analyses examined quality of life, unmet needs and sociodemographic factors associated with having a score of five or more on the Geriatric Depression Scale‐15.

**Results:**

Ninety‐four people participated in the study. Thirty‐seven percent (*n* = 35) reported some level of depressive symptoms, with most of these (21%, *n* = 20) classified as having mild depressive symptoms. Five participants (5%) reported they had had thoughts of being better off dead or hurting themselves, while three (3%) reported having had a plan to end their life. For each additional unmet need, the odds of being depressed increased by 25%(*P* < 0.001). For each point increase in quality of life, the odds of being depressed decreased by 48% (*P* < 0.001).

**Conclusions:**

The high proportion of people with dementia who report depressive symptoms suggests the need to routinely assess depressive symptoms among this group. There may also be benefits in assessing unmet needs and addressing these where possible as part of an approach to reducing depression among people living with dementia in the community.

## INTRODUCTION

Dementia is associated with loss of memory and cognitive function, as well as a range of neuropsychiatric symptoms. These may include agitation, depression, sleep disturbance, hallucinations, apathy and aggression.^1^ Neuropsychiatric symptoms are associated with higher levels of burden among care givers.^1^ Approximately 70% of people with dementia live in the community.^2^

A systematic review and meta‐analysis found that the mean prevalence of depression was 20% among people with Alzheimer's disease and 37% for people with frontotemporal lobe dementia, compared with 13% among similarly aged healthy controls.^3^ The majority of studies included in the review were conducted in North America. Of the five studies conducted in Australia, only four focussed on people living with dementia in the community, and only one of these was conducted in past the 10 years. This was a longitudinal study that explored neuropsychiatric symptoms among people living with dementia attending memory clinics. The prevalence of depression ranged between 49% at baseline to 41% at 3 years follow up.^4^ The 12‐item neuropsychiatric inventory was used to assess symptoms, including one item assessing depression. This tool is administered as a caregiver interview, and does not assess symptoms from the perspective of the person living with dementia.^5^

It is possible that prevalence of depression among community‐dwelling people with dementia is affected by changes in healthcare services or policy over time or by differences in how well depression is recognised and treated across settings and countries. Differences between proxy and self‐reported depression have also been reported.^6^ For this reason, it is important to obtain up‐to‐date information about depression among community‐dwelling people with dementia in Australia.

A number of studies have demonstrated a link between diagnosis of dementia and increased risk of suicide attempts, particularly early on in the course of the disease. For example, one nation‐wide matched cohort study from the United States of America (USA) found that those recently diagnosed with dementia were at increased risk of a suicide attempt compared to those without dementia.^7^ Few studies have examined risk of suicide or suicidal ideation among community‐dwelling people with dementia in Australia.

One review of prospective cohort studies found that people with Alzheimer's disease were more likely to develop depression if they had a history of psychiatric illness and were younger.^8^ Findings were mixed as to whether greater cognitive impairment and functional decline were associated with depression.^8^ Unmet needs have been shown to be associated with depression among community‐dwelling people with dementia.^9^, ^10^ A study by Eichler et al.^10^ was conducted in Germany and used an assessment of unmet needs that was compiled from a survey battery completed by the person with dementia or their caregiver. Black et al.^9^ conducted a study in the USA, and used a different measure of unmet needs from that used in the present study – the Johns Hopkins Dementia Care Needs Assessment.^11^ The latter is a clinician‐administered assessment rather than a self‐report assessment, and therefore, may not accurately capture the extent of unmet needs as perceived by the person with dementia. The current study focussed on the association between self‐reported unmet needs and depression.

### Aims

To explore, among a sample of community‐dwelling people with dementia in Australia,

1. the proportion who report normal, mild, moderate and severe depressive symptoms according to the Geriatric Depression Scale‐15 (GDS‐15), and the proportion who report thoughts of self‐harm or suicide, and
2. the association between sociodemographic variables, quality of life and unmet needs and reporting a depression score of greater than four on the GDS‐15.

## METHODS

### Design

This is a cross‐sectional survey of people with dementia living in the community. Human Research Ethics Committee Approval was obtained from Hunter New England Health (16/05/18/4.05; 17/07/19/4.06; 180 718/4.06) and the University of Newcastle (H‐2018‐0308). Data were collected between July 2018 and June 2020.

### Participants

Adults (aged 18 or older) who were diagnosed with dementia by a medical professional, able to read and write in English, and living in the community were eligible to participate. Those who did not have capacity to provide independent consent were excluded.

### Recruitment and data collection

Participants were recruited from settings in New South Wales and Victoria, Australia, including: geriatrician clinics (*n* = 10); respite centres for people with dementia (*n* = 3); support groups for carers of people with dementia (*n* = 3); and aged care providers (*n* = 2). Those identified as eligible were sent a survey pack consisting of a survey, study information and reply‐paid envelope. Where the information was available, the age and gender of those given a survey pack were recorded and used to assess consent bias. This information was not available for the carer support groups or respite centres.

Completion of the survey was taken as implied consent. Capacity to consent was assessed using four questions presented at the start of the survey. These questions were adapted from the University of California, San Diego Brief Assessment of Capacity to Consent (UBACC).^12^ Participants were asked to select the correct response from three possible response options. Those who answered at least three out of four questions correctly were classified as having adequate capacity to consent, in alignment with the cut‐off of 14.5 out of 20 on the original UBACC measure.

### Measures

#### Sociodemographic and health characteristics

Participants were asked to self‐report their age, sex, and who they lived with. Participants were also asked, ‘Do you have any chronic health conditions?’ They were asked to tick ‘no’ or select as many as applied from the following: cancer, heart disease, lung disease, diabetes, arthritis, back pain, mental health condition (e.g., depression) or ‘other’. If the person had a carer, carers were asked to report on the type of dementia the person had, time since diagnosis, and rate the severity of their dementia symptoms from 1 to 10.

#### Depression

The GDS is a screening tool specifically designed for use with older adults.^13^ It has been shown to be a reliable and valid tool for assessing depression, including among people with mild to moderate dementia.^14^ The scale includes 15 items assessing depressive symptoms in the past week using a yes/no response scale. Each ‘yes’ response is scored as one point. Scores are summed across the 15 items to give a total score. Scores of four and below are considered normal while a score 5–8 is indicative of mild depression, 9–11 of moderate depression and 12–15 of severe depression.^15^

#### Thoughts of suicide or self‐harm

Participants were asked about thoughts of suicide or self‐harm in the past week using two items. One item was adapted from a question included in the Patient Health Questionnaire‐9:^16^ ‘Have you thought that you would be better off dead or that you want to hurt yourself in some way?’; while the second item was developed for this study: ‘Have you thought about a plan to end your life?’. A yes/no response scale was used.

#### Quality of life

A 10‐point visual analogue scale was used to assess global quality of life. Participants were asked, ‘Overall how would you rate your own quality of life?’ The anchor points 1 = ‘very poor’ and 10 = ‘excellent’ were used.

#### Unmet needs

The Unmet Needs – Dementia (UNI‐D) scale was used to assess perceived need for help across the following domains: daily tasks (eight items), activities and hobbies (four items), health and wellbeing (five items), access to support (four items), and getting support for dementia (five items).^17^ Respondents were presented with 26 items beginning with the stem, ‘Do you need ….’ with a yes/ no response scale. Affirmative responses were summed to provide a total unmet need score.

### Statistical analysis

Descriptive statistics were calculated for categorical variables including demographics, depression categories, and thoughts of suicide or self‐harm.

Consistent with GDS‐15 scoring guidelines, participants with up to 20% missing responses^18^ (three missing responses) on the GDS‐15 had their scores imputed.^19^ Participants (*n* = 1) with more than three missing responses on the GDS‐15 were excluded from analysis.

The association between depression (GDS score >4) and the factors of interest (age, gender, presence of chronic illness (other than dementia), unmet needs count, and quality of life score) were modelled using univariable logistic regressions. All factors were then included in a single multivariable logistic regression. Estimates are expressed as odds ratios (ORs), modelling the odds of being depressed.

The assumption of linearity for the two numeric variables, unmet needs count and quality of life, was checked by plotting the log odds of the outcome against them. Statistical analyses were conducted using SAS v.9.4.

## RESULTS

A total of 316 people with dementia were sent an information pack. Of these 112 returned a completed survey (response rate 35%). No evidence of response bias was found (gender *P* = 0.07, *n* = 186; age *P* = 0.87, *n* = 172). Six of those who responded scored lower than four on the capacity questions and seven missed these questions. These people were excluded to ensure the responses were reliable. A further four were excluded as they lived in a residential aged care facility. The final sample consisted of 94 participants. Characteristics of the sample are shown in Table 1. The majority of participants were male (60%), aged at least 75 years (79%), lived with their partner or spouse (82%) and had at least one additional health condition (73%). Sixty‐five of the participants had a carer who reported on their type of dementia, time since diagnosis and symptom severity. Based on carer report, 51% had Alzheimer's disease and 60% had been diagnosed in the last 3 years. Carers' mean rating of symptom severity was 5. 3 (SD = 1.7) out of 10 (*n* = 61).

**Table 1:** Demographic characteristics of participants

Variable	Variable level	*n*	%
Age (*n* = 90)	55–75	19	21%
	75–85	49	54%
	85–100	22	24%
Gender (*n* = 93)	Male	56	60%
	Female	37	40%
Dementia type (*n* = 63)^†^	Alzheimer's disease	32	51%
	Vascular dementia	6	9.5%x
	Other	10	16%
	Do not know	15	24%
Time since diagnosis (*n* = 60)^†^	Less than 3 years	36	60%
	More than 3 years	24	40%
Living arrangement (*n* = 94)	Alone	6	6%
	Partner/spouse	77	82%
	Child/grandchild	11	12%
Multimorbidity (*n* = 90)	Yes	66	73%
	No	24	27%
Depression category (GDS‐15; *n* = 94)	Normal	59	63%
	Mild	20	21%
	Moderate	12	13%
	Severe	3	3.2%

Variable	Variable level	Mean	SD
Age (*n* = 90)	Years	79.3	8.0
Symptom severity (*n* = 61)^†^	Scale (1–10)	5.3	1.7
Quality of life (*n* = 91)	Scale (1–10)	7.5	1.7
Unmet needs (*n* = 94)	Scale (0–26)	5.6	5.1
GDS‐15 score (*n* = 94)	Scale (0–15)	3.9	3.5

^†^ Participant's informal carer where available, was asked this information.

Abbreviation: GDS‐15, Geriatric Depression Scale‐15.

### Proportion of participants classified as depressed and as having thoughts of self‐harm/suicide

The majority of participants were classified as not depressed (*n* = 59, 63%), 20 (21%) were classified as having mild depression, 12 (13%) as having moderate depression and three (3%) as having severe depression.

For the item, ‘Have you thought that you would be better off dead or that you want to hurt yourself in some way?’ five (6%) people responded ‘yes’, and six people (6%) did not respond. For the item, ‘Have you thought about a plan to end your life?’ three (3%) people responded ‘yes’ and three (3%) did not respond to this question.

Of the five people who reported thinking they would be ‘better off dead’, three were rated as having mild depression (GDS scores of five, seven and eight), one as having moderate depression (GDS score nine), and one as having severe depression (GDS score 13). Of the three who reported that they had ‘thought about plan to end life’, one was rated as severely depressed (GDS score 13), one as moderately depressed (GDS 10) and one as mildly depressed (GDS score eight).

Only one person, rated as having severe depression, endorsed both ‘thought that would be better off dead’ and ‘thought about plan to end life.’ This person also rated their quality of life as poorer than the average for the sample (4/10) but reported fewer unmet needs than average (two unmet needs).

Six out of the seven participants endorsing thoughts of self‐harm reported higher than average numbers of unmet needs for the entire sample, and five out of the seven reported lower than average quality of life. Carer ratings of symptom severity were only available for four of the participants; however, all four had ratings that were higher than average for the sample (6–7 out of 10).

### Exploring relationship between variables of interest and depression

The unadjusted odds of being depressed (OR = 1.34) increased by 34% for each additional unmet need reported (*P* < 0.001). For each point increase in quality of life, the unadjusted odds of being depressed decreased by 55% (*P* < 0.001). None of the other variables demonstrated a significant relationship with depression in the bivariate analyses.

In the multivariable analyses, unmet needs and quality of life remained significantly associated with depression (Table 2). For each additional unmet need, the odds of being depressed increased by 25% (*P* < 0.001). For each point increase in quality of life the odds of being depressed decreased by 48% (*P* < 0.001).

**Table 2:** Multivariable analyses showing adjusted odds ratios associated with depression

Variable	Variable level	Odds ratio (95% CI)	*P*‐value	Overall *P*‐value	Number of observations
Age	<75	Reference		0.505	85
	75–85	1.01 (0.23, 4.42)	0.989		
	>85	2.29 (0.40, 13.14)	0.354		
Gender	Male	Reference			
	Female	0.79 (0.24, 2.61)	0.701		
Other chronic health conditions	No	Reference			
	Yes	0.73 (0.19, 2.75)	0.639		
Unmet needs count quality of life		1.25 (1.09, 1.44)	<0.001		
		0.52 (0.34, 0.77)	<0.001		

To examine whether there was a difference in the types of needs experienced by those scoring below and above the threshold for depression on the GDS‐15 scale, we performed χ^2^ tests of association for each of the 26 needs items. After applying a Bonferroni correction for multiple comparisons, eight items showed a significant difference. Needs that were more likely to be endorsed by people with depression are shown in Table 3. Five out of the seven needs items showing a significant relationship with depression were related to needing help with engagement in meaningful activities (e.g., ‘support to do things by yourself’, ‘activities you enjoy’) and maintaining social connections (‘to see friends and family more often’). The remaining two items reflected unmet needs for help with finding information: ‘understanding who you should contact if you have a problem or concern related to dementia’ and ‘understanding what dementia is’.

**Table 3:** Unmet needs which are significantly more likely to be reported by people scoring above threshold on the Geriatric Depression Scale‐15 compared to those scoring below threshold for depression

Unmet need	Not depressed *n* (%)	Depressed *n* (%)	Chi^2^	Fisher's exact test
Support to do things by yourself	8 (14%)	18 (51%)	15.7	**<0.001**
To see friends and family more often	11 (19%)	20 (57%)	14.7	**<0.001**
Feelings that you have been experiencing	5 (8%)	23 (66%)	34.4	**<0.001**
Understanding who you should contact if you have a problem or concern related to dementia	13 (22%)	21 (60%)	13.7	**<0.001**
Gardening	8 (14%)	16 (46%)	11.9	**0.001**
Activities you enjoy	7 (12%)	15 (43%)	11.8	**0.001**
Maintain routines of things you have always done	3 (5%)	11 (31%)	12	**0.001**
Understanding what dementia is	8 (14%)	16 (46%)	11.9	**0.001**

*Note*: Bold indicates significant value.

## DISCUSSION

Depression is recognised as a symptom of dementia, therefore the high proportion of our sample (37.2%) that were classified as experiencing depression is unsurprising. Encouragingly most reported mild levels of depressive symptoms and few reported suicidal thoughts. Reviews have reported rates of depression between 15.9% for all‐cause dementia, 14.8%^20^ to 20% for Alzheimer's disease and 37% for frontotemporal lobe dementia.^3^ The variability in reported rates may be due to methodological differences in the measurement of depression, and differences in sample composition by dementia sub‐type, severity and setting (residential care versus community).

The proportion of participants reporting suicidal or self‐harm ideation was lower than in previous studies.^21^, ^22^ This may reflect differences across study samples such as dementia progression and dementia sub‐type or methods of assessment of this sensitive issue. The small proportion of missing data for these questions (7%) suggests that people with dementia are comfortable with answering questions regarding suicide and self‐harm. Only one person who reported having feelings that they would be better off dead also reported having a plan to end their life. This, together with the severe depression reported by this individual, suggests that they were at higher risk of suicide than other participants. All of those endorsing the self‐harm items reported some level of depressive symptoms, and most reported poorer quality of life and greater unmet needs than average for this sample. While limited by the small numbers, this may indicate the importance of addressing unmet needs, treating depression and slowing decline in quality of life as much as possible.

Identifying depression among people with dementia is complex, as the two conditions present common symptoms, including apathy, withdrawal from social activities, and loss of interest in usual activities. Use of validated screening measures such as the GDS‐15 may assist in disentangling major depressive disorder from dementia.^23^ Management of depression among people with dementia is limited by a lack of evidence for effective therapeutic strategies. A Cochrane review found little evidence to support the efficacy of antidepressant medications for the treatment of depression in dementia.^24^ There is evidence from a review of systematic reviews to support the effectiveness of psychological therapies such as cognitive behavioural therapy in the treatment of depression among people living with dementia.^25^ However, it was noted that further research is needed to identify the most effective non‐pharmacological treatments for specific sub‐types of dementia. In Australia, a limited number of sessions for psychological therapies can be accessed through Medicare with a general practitioner referral. A rebate from Medicare, Australia's universal health cover system, covers a portion of the cost; however, there is still often an out‐of‐pocket expense to the client. This may serve as a barrier to access.

We found that poorer quality of life and number of unmet needs are associated with greater depression scores. This accords with other research that has shown that poor quality of life is associated with depression among people with living with dementia.^26^ Similar to our results, a US study found that depressive symptoms were associated with higher unmet needs among people with dementia.^9^ As with quality of life, the direction of causality of this relationship cannot be determined by our study. However, it is plausible that this is also bi‐directional. Having less support may contribute to the onset of depression. Conversely, people with depression may have greater needs for support and thus have more of these needs unmet. This may suggest that unmet needs for support should be identified and addressed in tandem with depressive symptoms.

Five out of the seven unmet needs that were more prevalent among people with depression than those without related to maintaining activities and social connections. This aligns with other research that being able to engage in meaningful activity is important to quality of life.^27^ This suggests that being able to support people with dementia to keep engaged in activities and social activities where possible, may be worth investigating as a potential therapeutic strategy. Conversely, it may also simply reflect that people who are depressed tend to disengage from activities. Longitudinal and experimental data are needed to tease out these relationships.

Our study found that having other chronic conditions was not associated with increased likelihood of depression. Reviews have shown that depression is more likely to occur among people with multimorbidity than among people with no chronic conditions.^28^ A large Australian study of over 7000 primary care patients found that risk of depression varied by chronic disease type and increased slightly as the number of comorbidities increased.^29^ A Swedish prospective cohort study of patterns of multimorbidity among older people found that dementia and depression co‐occurred more commonly than would be expected by chance.^30^ Therefore, it is possible that in the current study, the small sample size together with selection bias, resulted in the failure to identify a link between comorbidity and depression. Alternatively, it is possible that any associations between particular chronic diseases and depression were obscured by the risk of depression conferred by a diagnosis of dementia.

### Limitations and future directions

The small sample size and modest response rate limit generalisability of the results. As participants were recruited through varied sources it was not possible to confirm sub‐type of dementia in this study via clinician report or medical record audit. This may be a limitation, given evidence that prevalence of depression may vary according to dementia sub‐type.^3^ Further, the single item used by carers to rate perceived severity of dementia has not been validated and may have resulted in dementia severity being under‐estimated.^31^

The study‐specific measures of quality of life and suicidal ideation could undergo testing to determine validity and reliability among people with dementia. Our study should be replicated with a larger sample. Longitudinal studies may also tease apart the causal relationships between unmet needs, quality of life and depression. Intervention studies which seek to address unmet needs and quality of life, may also be examined for their impact on depression. This would provide valuable information about the range of interventions that can be used to target depression and the causal relationship among these variables.

## CONCLUSIONS

Rates of self‐reported depressive symptoms are high among people living with dementia in the community. A greater number of reported unmet needs increases the odds of reporting depression; while better self‐reported quality of life is associated with lower odds of depression. These results highlight the importance of identifying and managing both symptoms of depression and unmet needs among people living with dementia.

## DISCLOSURE

Authors declare no conflict of interests for this article.

## Data Availability

The data that support the findings of this study are available on request from the corresponding author. The data are not publicly available due to privacy or ethical restrictions.
